# The Use of Social Media on Enhancing Dental Care and Practice Among Dental Professionals: Cross-Sectional Survey Study

**DOI:** 10.2196/66121

**Published:** 2025-01-03

**Authors:** Joseph Macadaeg Acosta, Palinee Detsomboonrat, Pagaporn Pantuwadee Pisarnturakit, Nipaporn Urwannachotima

**Affiliations:** 1 International Graduate Program in Dental Public Health, Department of Community Dentistry, Faculty of Dentistry, Chulalongkorn University, Bangkok, Thailand; 2 School of Dentistry, University of Baguio, Baguio City, Philippines; 3 Faculty of Dentistry, Department of Community Dentistry, Chulalongkorn University, 34 Henri Dunant Road, Pathumwan, Bangkok, 10330, Thailand, 66 02-2188543

**Keywords:** social media, oral health promotion, oral health education, dentists, dental practice, dental professionals, dental practitioners

## Abstract

**Background:**

As digitalization continues to advance globally, the health care sector, including dental practice, increasingly recognizes social media as a vital tool for health care promotion, patient recruitment, marketing, and communication strategies.

**Objective:**

This study aimed to investigate the use of social media and assess its impact on enhancing dental care and practice among dental professionals in the Philippines.

**Methods:**

A cross-sectional survey was conducted among dental practitioners in the Philippines. The study used a 23-item questionnaire, which included 5 questions on dentists’ background and demographic information and 18 questions regarding the use, frequency, and purpose of social media in patient advising and quality of care improvement. Data were analyzed using SPSS software, with frequency distributions and *χ*^2^ tests used to assess the association between social media use and demographic variables and the impact on dental practice.

**Results:**

The 265 dental practitioners in this study were predominantly female (n=204, 77%) and aged between 20‐30 years (n=145, 54.7%). Most of the participants were general practitioners (n=260, 98.1%) working in a private practice (n=240, 90.6%), with 58.5% (n=155) having 0‐5 years of clinical experience. Social media use was significantly higher among younger practitioners (20‐30 years old) compared to older age groups (*P*<.001), though factors such as sex, dental specialty, and years of clinical practice did not significantly influence use. The majority (n=179, 67.5%) reported using social media in their practice, primarily for oral health promotion and education (n=191, 72.1%), connecting with patients and colleagues (n=165, 62.3%), and marketing (n=150, 56.6%). Facebook (n=179, 67.5%) and YouTube (n=163, 61.5%) were the most frequented platforms for clinical information, with Twitter (subsequently rebranded X) being the least used (n=4, 1.5%). Despite widespread social media engagement, only 8.7% (n=23) trusted the credibility of web-based information, and 63.4% (n=168) perceived a potential impact on the patient-dentist relationship due to patients seeking information on the internet. Social media was also perceived to enhance practice quality, with users reporting significant improvements in patient care (*P*=.001).

**Conclusions:**

The findings highlight that social media is widely used among younger dental practitioners, primarily for education, communication, and marketing purposes. While social media use is associated with perceived improvements in practice quality and patient care, trust in information on social media remains low, and concerns remain regarding its effect on patient relationships. It is recommended to establish enhanced guidelines and provide reliable web-based resources to help dental practitioners use social media effectively and responsibly.

## Introduction

Traditionally, dental practices advertised their services primarily through local yellow pages, such as telephone directories listing local businesses or by placing promotional notices in the windows of dental offices [1]. Word-of-mouth was also a common method, where information about the quality of service was shared from one person to another [2]. However, with the advent of technological advancements, there has been a significant shift toward the use of social media. Social media platforms offer the potential to reach diverse audiences, including those seeking information on oral health management, thereby promoting oral health [3].

Social media refers to a group of web-based applications built on the foundations of Web 2.0 [4,5]. According to the Merriam-Webster Dictionary, social media is defined as “any form of electronic communication through which users create web-based communities to share information, personal messages, ideas, and other content such as photos and videos” [6]. Popular platforms include social networking sites like Facebook and Twitter (subsequently rebranded X), media-sharing sites like YouTube and Instagram, blogs, and microblogging sites [7]. These platforms are widely used for searching, sharing, and communicating information, including health-related content [8,9]. Facebook fosters connections through its friend-based network, creating a web-based community where users can stay in touch and share content [10]. Twitter’s microblogging feature allows users to share ideas and opinions in real time [11,12]. YouTube is a leading video-sharing platform [13-15], while Instagram, a photo and video social networking site, facilitates real-time communication through comments and direct messages and supports professional engagement and knowledge sharing [16-18]. TikTok, known for its short video content, has also gained popularity [19,20].

Social media serves as a dynamic tool for exchanging information and communicating with colleagues, patients, and the public on health-related issues. Public health professionals and organizations use these platforms for a variety of purposes, including health education, telemedicine, social marketing, scientific research, recruitment, career development, and professional networking [9]. Dental practitioners are increasingly leveraging social media to educate and inform the public by sharing detailed content on various dental procedures and educational activities [8,21-25], providing oral hygiene instructions and promoting oral health [26,27], enhancing communication [28-30], and engaging in marketing efforts [1,25,31,32]. Given the widespread use of social media, using these platforms responsibly and effectively in health care is crucial to ensure that they contribute positively to patient health outcomes.

As of April 2024, there were 5.44 billion internet users worldwide, representing 67.1% of the global population. Of this total, 5.07 billion, or 62.6% of the world’s population, were social media users [33]. Based on the number of monthly active users, the most popular social networks globally are Facebook, YouTube, Instagram, WhatsApp, and TikTok [34]. In the Philippines, approximately 68.72 million people are social media users, with an average daily use of over 3 hours, the highest across the Asia-Pacific region. Facebook, Instagram, TikTok, and X (formerly known as Twitter) are currently some of the most popular platforms, providing opportunities for connecting with family and friends, sharing content via digital platforms, and expanding the reach of promotional marketing through web-based banners [35]. Social media in the Philippines has gained significant traction for entertainment, communication, marketing, and professional education. In dentistry, practitioners use these platforms to share content related to oral health, modern treatment options, trends in dental materials, and treatment costs.

Although several reports have highlighted the use of social media among dentists and its influence on dental practice [3,8,9,22,30,32,36,37], as well as its impact on dental education among faculty and students [7,21,23,38,39], relatively few studies have examined the use and influence of social media on dental practice in Southeast Asia, particularly in the Philippines where these platforms are widely used. This study aims to investigate the use of social media and assess its impact on enhancing dental care and practice among dental professionals in the Philippines.

## Methods

### Design and Study Population

This study used a cross-sectional survey design with purposive sampling, targeting all eligible licensed dental professionals, including both general dentists and specialists working in private or public clinical settings and who were involved in patient care, education, and practice management. Participants were also required to be active members of the Philippine Dental Association-Baguio City Chapter for the 2023‐2024 term. The study population consisted of 265 dental practitioners. Practitioners who were not active members of the chapter were excluded from the study.

The survey questionnaire was adapted from previously published literature related to social media use [22,36,38]. For this study, social media included popular platforms such as Facebook, YouTube, Twitter, Instagram, TikTok, and Viber, which dental practitioners may have used for professional purposes like patient education, practice marketing, peer communication, and access to clinical information. A panel of experts validated the questionnaire items and the questionnaire underwent pretesting to ensure applicability and reliability. Revisions and minor adjustments were made based on the pilot study results and expert feedback. The 23-item questionnaire was divided into two sections. The first section gathered background and demographic information, including age, sex, dental specialty, work sector, and years of clinical practice. The second section comprised 18 questions designed to gather information on social media use; frequency of use; and purpose in dental practice, particularly its role in advising patients and improving the quality of care. This study was conducted within dental practice settings, with participants recruited through web-based methods. Recruitment emphasized voluntary participation, with all respondents providing informed consent before inclusion in the study. The questionnaire was administered via Google Forms, with the survey link disseminated through email, a Facebook page, and the Baguio City Chapter group chat. Respondents were encouraged to provide suggestions, assist in recruitment, and further share the questionnaire with other dental practitioners to reach more participants. They were given one day to complete the form.

### Data Analysis

Data were analyzed using SPSS software version 28.0 (IBM Corp). The analysis included frequency distributions that were expressed in numbers and percentages, as well as *χ*^2^ tests to assess the association between social media use and demographic or social variables and the impact on dental practice. Statistical significance was set at an α level of .05. This study adhered to the reporting guidelines for cross-sectional studies following the Strengthening the Reporting of Observational Studies in Epidemiology (STROBE) statement.

### Ethical Considerations

Ethical approval was obtained from the Human Research Ethics Committee of the Faculty of Dentistry, Chulalongkorn University (Study Code: HREC-DCU 2023‐108). Informed consent was obtained from all participants through a written consent form, which provided details about the study’s objectives and potential benefits. Participation in the study was entirely voluntary, with participants having the right to withdraw or discontinue at any time without any penalties or loss of benefits. All information was kept strictly confidential and anonymized to ensure participants could not be identified. No financial rewards or incentives were provided for participation.

## Results

The background and demographic details of the 265 study participants are summarized in Table 1. The majority of participants were female (n=204, 77%), with over half (n=145, 54.7%) aged between 20 to 30 years. This was followed by participants aged 31‐40 years (n=52, 19.6%), and both groups of participants aged 41‐50 years and over 50 years represented 12.8% (n=34) of participants. Most participants (n=260, 98.1%) were general practitioners, and 90.6% (n=240) worked as private dentists. Regarding clinical experience, 58.5% (n=155) had 0 to 5 years of practice, while 29.8% (n=79) had over 10 years, and 11.7% (n=31) had 6 to 10 years of practice.

**Table 1.** Demographic variables and their association with the use of social media among dental practitioners.

Variable	Use of social media, n (%)^a^			Total (n=265), n (%)	*P* value^b^
	Yes	Sometimes	No		
Age (years)					<.001
20‐30	102 (70.3)	35 (24.1)	8 (5.5)	145 (54.7)	
31‐40	31 (60)	21 (40)	0 (0)	52 (19.6)	
41‐50	27 (79)	2 (6)	5 (15)	34 (12.8)	
>50	19 (56)	15 (44)	0 (0)	34 (12.8)	
Sex					.66
Male	40 (66)	19 (31)	2 (3)	61 (23)	
Female	139 (68.1)	54 (26.5)	11 (5.4)	204 (77)	
Dental specialty					.25
General practitioners	177 (68.1)	70 (26.9)	13 (5)	260 (98.1)	
Specialists	2 (40)	3 (60)	0 (0)	5 (1.9)	
Working sector					.30
Government	10 (71)	2 (14)	2 (14)	14 (5.3)	
Private	160 (66.6)	69 (28.8)	11 (4.6)	240 (90.6)	
Both (government and private)	9 (82)	2 (18)	0 (0)	11 (4.2)	
Number of years of clinical practice					.19
0 to 5	109 (70.3)	38 (24.5)	8 (5.2)	155 (58.5)	
6 to 10	24 (77)	7 (23)	0 (0)	31 (11.7)	
>10	46 (58)	28 (35)	5 (6)	79 (29.8)	

a Percentages were calculated based on the number of participants corresponding to each variable.

b Chi-square test; *α*=.05.

There was a significant increase in social media use among the younger age group (20‐30 years) compared to older age groups (31‐40 years, 41‐50 years, and >50 years), with a *P *value of <.001. However, sex, dental specialty, work sector, and years of clinical practice did not significantly influence social media use.

Table 2 illustrates the frequency distribution of social media use among dental practitioners. Of the 265 participants, most reported moderate use of social media with 37.4% (n=99) using it 1‐2 days per week. However, nearly two-thirds (n=164, 61.9%) reported using social media at least 3 days weekly. Facebook (n=179, 67.5%) and YouTube (n=163, 61.5%) were the most commonly visited platforms for obtaining clinical information, followed by Instagram (n=115, 43.4%). Twitter (n=4, 1.5%) was the least popular platform. Social media was mainly used for oral health education and promotion (n=191, 72.1%); communication with patients, friends, and family (n=165, 62.3%); and marketing and advertising (n=150, 56.6%).

**Table 2.** Frequency, preferred platforms, and purpose of social media use.

Variables	Frequency (n=265), n (%)
Social media use	
Never	2 (0.8)
Seldom (average of 1‐2 d/wk)	99 (37.4)
Occasionally (average of 3‐5 d/wk)	96 (36.2)
Frequently (average of 6‐7 d/wk)	68 (25.7)
Social media website used^a^	
Twitter	4 (1.5)
Facebook	179 (67.5)
Instagram	115 (43.4)
YouTube	163 (61.5)
TikTok	38 (14.3)
Viber	23 (8.7)
Others	75 (28.3)
Purpose of social media use^a^	
Oral health promotion and education	191 (72.1)
Connect and communicate with patients, friends, and family	165 (62.3)
Exchange opinions and views regarding cases with colleagues	113 (42.6)
Marketing and advertising	150 (56.6)

a Given that study participants could indicate multiple responses, the sum of the percentages is not 100.

**Figure 1.**
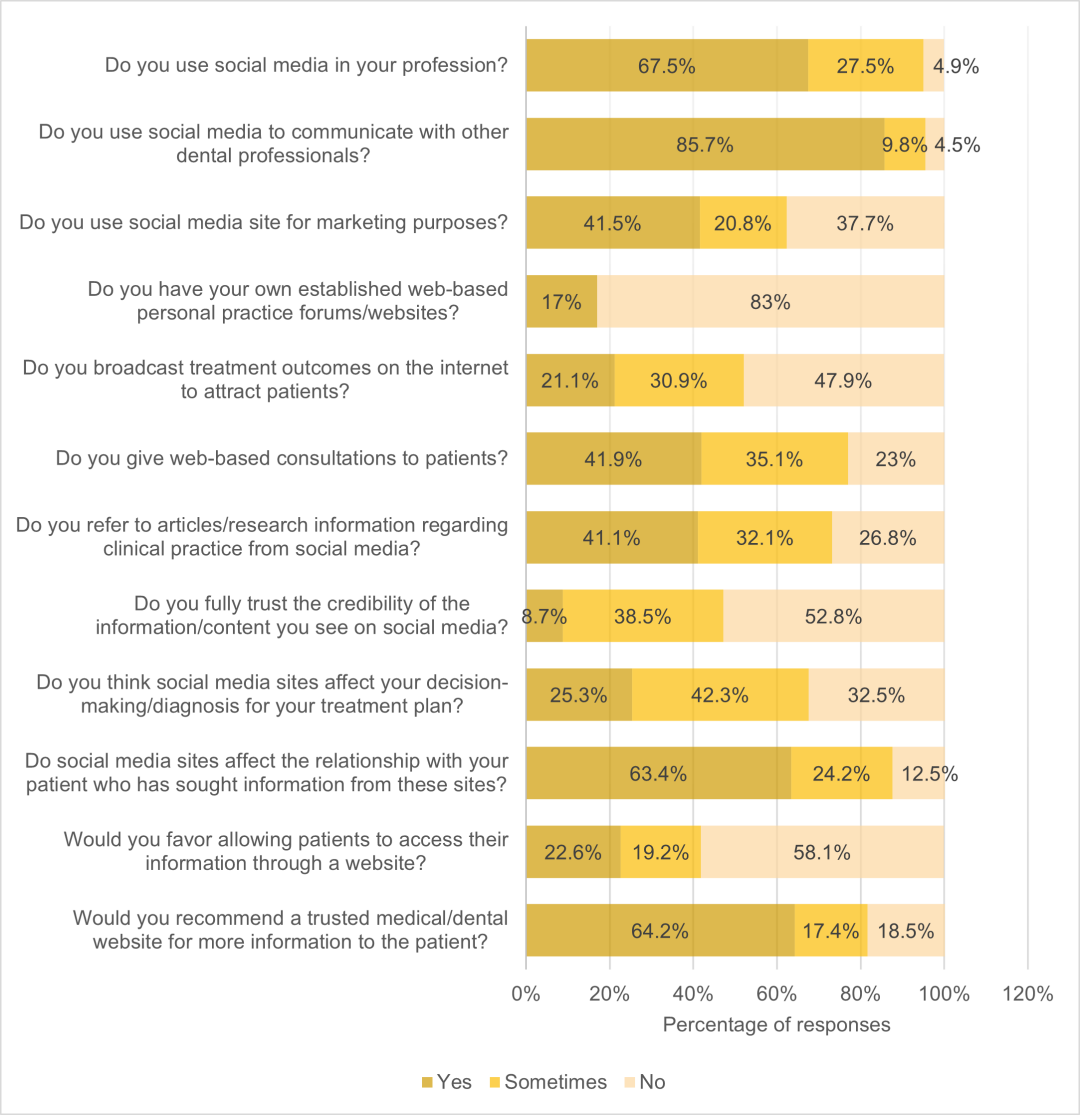
Percentage of dental practitioners’ responses to questions on social media use (n=265).

The responses of dental practitioners to questions regarding social media use are presented in Figure 1. Of the 265 participants, 67.5% (n=179) indicated that they use social media in their dental practice. Despite this, 83% (n=220) of dentists did not have their own web-based practice forums or websites. However, the majority (n=227, 85.7%) reported using social media to communicate with other dental professionals, and 41.5% (n=110) used it for marketing purposes. Although 41.1% (n=109) of dentists referred to articles or research information from social media for clinical practice, nearly half (n=127, 47.9%) were reluctant to broadcast treatment outcomes on the internet to attract patients, and 41.9% (n=111) were open to providing web-based consultations. Only 8.7% (n=23) of dentists trusted the credibility of information on social media, while 63.4% (n=168) believed that social media can affect the patient-dentist relationship when patients seek information on the internet. Additionally, most participants (n=154, 58.1%) did not allow patients to access their information through a website, but 64.2% (n=170) would recommend a trusted website to patients.

Table 3 explores the relationship between social media use and its impact on dental practice. Participants who used social media in their practice believed that it enhanced the quality of care provided to patients (*P*=.001) and significantly reported that it improved their dental practice (*P*=.02).

**Table 3.** Use of social media and respondents’ perceived impact on enhancing patient care quality and dental practice.

Variable	Use of social media, n (%)^a^			Total (n=265), n (%)		*P* value^b^
	Yes	Sometimes	No			
Social media improves the quality of care delivered to patients						.001
Yes	126 (74.1)	40 (23.5)	4 (2.4)	170 (64.2)		
Maybe	42 (56)	24 (32)	9 (12)	75 (28.3)		
No	11 (55)	9 (45)	0 (0)	20 (7.5)		
Social media improves dental practice						.02
Yes	106 (74.7)	30 (21.1)	6 (4.2)	142 (53.6)		
Maybe	48 (57)	29 (35)	7 (8)	84 (31.7)		
No	25 (64)	14 (36)	0 (0)	39 (14.7)		

a Percentages were calculated based on the number of participants corresponding to each variable.

b Chi-square test was used with an *α* level of .05.

## Discussion

### Principal Findings

This study aimed to investigate the use of social media and assess its impact on enhancing dental care and practice among dental professionals. The findings revealed that over half of the participants aged 20‐30 years are actively using social media in their dental profession, aligning with other studies that indicate younger dentists or health care professionals are more likely to use social media compared to their older counterparts [36,38,40-42]. Variables such as sex, dental specialty, work sector, and years of clinical practice did not significantly influence social media use.

Most respondents reported using social media 1‐2 days per week to engage with the public and other dental professionals. This is in contrast to other studies where social media was used at least once daily for obtaining information [32,42]. Among social media users in the Philippines, Facebook and YouTube were identified as the primary platforms for accessing clinical information, which is consistent with The Digital Report 2024, highlighting that Facebook has 86.75 million active users in the Philippines [43]. These findings are similar to those reported among dentists in the United Kingdom and the United States, where Facebook is the most commonly used platform in dental practice [1,31]. This was also true as among dental students [7]. In comparison, dentists in Saudi Arabia predominantly use Twitter [22,25] and Instagram [32], while WhatsApp is the preferred platform among Indian dentists [37]. Similarly, South African dentists primarily use Google Plus and Facebook for personal rather than professional purposes [42].

Previous studies have shown that marketing is a common reason for using social media [1,25,31,32]. In our study, 72.1% (191/265) of participants indicated that their primary reason for using social media was for oral health promotion and education. This finding is consistent with earlier research, which found that a high proportion of respondents use social media for dental education and prefer to share clinical work [21]. Other studies also support the notion that education and sourcing oral health information are primary purposes for social media use among dental professionals [22-25]. Social media interventions have positively impacted various aspects of oral health, particularly among adolescents, where platforms like YouTube, WhatsApp, Instagram, and Telegram have been effectively used to promote oral health [27]. For instance, a study demonstrated that oral health education via Telegram significantly improved oral health outcomes among secondary school students [44] and adolescents [45] in Iran. Additionally, Instagram has been shown to support orthodontists in motivating young patients to maintain proper oral hygiene [46], and WhatsApp has been integrated successfully into oral hygiene protocols to enhance patient compliance and oral health during orthodontic treatment [47]. Presenting audiovisual information through YouTube has also significantly improved patient knowledge [48].

Another key purpose of social media use is communication and the exchange of opinions with colleagues. According to the survey, 62.3% (165/265) of participants used social media to connect with patients, friends, and family, while 85.7% (227/265) used it to communicate with other dental professionals. This is supported by studies showing that social media communication, such as Instagram, can reduce patient anxiety prior to dental procedures [28], enhance dentists’ communication skills with other professionals [38], and improve patient interactions [30]. Another study found that a significant proportion of the younger population engages with social media to discuss dentistry. This highlights the need for careful management to ensure the dissemination of accurate dental health information [25].

The respondents of the survey also indicated that social media had the potential to enhance dental practice and the quality of care provided to patients. This finding is in agreement with study that shows that dentists who use social media in their practice not only recommend it to their peers but also believe it enhances their practice [22]. Social media can improve various aspects of dental practice, including service provision, advertising, counseling, and oral health education, and it can also be a tool for professional development [49]. Dental organizations and educators can use social media to disseminate information and updates [36,50]. Furthermore, multidimensional health care approaches, including social media, have been proven highly successful in improving patient care, increasing public knowledge, facilitating research, connecting health care providers, improving medical education, and addressing public health crises [29].

This study has several limitations that should be considered when interpreting the results. First, the study population was limited to dental practitioners in Baguio City, Philippines, which may restrict the generalizability of the findings to the broader dentist population in the country. The uneven distribution of survey participation across various demographic variables, such as age, sex, dental specialty, working sector, and years of clinical practice, may further impact the ability to extrapolate the results to the entire dental community. Additionally, the use of an electronic survey as the sole data collection method presented inherent challenges. A significant portion of the target population may not respond to or use electronic surveys, potentially introducing nonresponse bias and affecting the representativeness of the sample. This bias could be particularly relevant in the context of social media use, as those who are less inclined to participate in electronic surveys may also be less likely to engage with social media platforms. Furthermore, the cross-sectional nature of the study limits the ability to establish causal relationships between social media use and its impact on dental practice and patient care. Longitudinal studies would be necessary to better understand the long-term effects of social media integration in dental settings. Despite these limitations, this study provides valuable insights into the current use of social media among dental practitioners in the Philippines and its perceived impact on dental care and practice. Future research should aim to include larger, more representative samples across different regions and settings to enhance the generalizability of the findings. Additionally, mixed-methods approaches combining quantitative and qualitative data collection techniques could provide a more comprehensive understanding of the topic.

### Conclusions

Social media is widely used among younger dental practitioners, primarily for education, communication, and marketing, with a notable impact on enhancing practice quality and patient care. Social media platforms, especially Facebook and YouTube, were commonly used by practitioners to promote oral health, engage with patients and colleagues, and support marketing efforts.

Social media offers a convenient space for professional growth, patient education, and community interaction, which many dentists perceive as beneficial to their practice. However, a low trust in social media information and concerns about its influence on patient-dentist relationships indicate the need for clear guidelines and quality web-based resources. Enhanced support in these areas can help dental professionals maximize the positive impacts of social media on dental care and practice.

Future studies should compare social media with traditional marketing methods in the dental practice such as print ads, community outreach, and patient referrals. These traditional methods remain valuable for building trust, local credibility, and reaching offline patients. Comparing these strategies can help find the best way to combine digital and traditional approaches for better patient engagement and practice success.

## Abbreviations

STROBE: Strengthening the Reporting of Observational Studies in Epidemiology
